# Integrated bioinformatics approach reveals methylation-regulated differentially expressed genes in obesity

**DOI:** 10.20945/2359-3997000000604

**Published:** 2023-05-10

**Authors:** Guilherme Coutinho Kullmann Duarte, Felipe Pellenz, Daisy Crispim, Tais Silveira Assmann

**Affiliations:** 1 Hospital de Clínicas de Porto Alegre Porto Alegre RS Brasil Serviço de Endocrinologia, Hospital de Clínicas de Porto Alegre, Porto Alegre, RS, Brasil; 2 Universidade Federal do Rio Grande do Sul Faculdade de Medicina Porto Alegre RS Brasil Programa de Pós-graduação em Ciências Médicas: Endocrinologia, Faculdade de Medicina, Universidade Federal do Rio Grande do Sul, Porto Alegre, RS, Brasil

**Keywords:** Bioinformatics, obesity, methylation-regulated differentially expressed genes (MeDEGs), PPI network

## Abstract

**Objective:**

To identify DNA methylation and gene expression profiles involved in obesity by implementing an integrated bioinformatics approach.

**Materials and methods:**

Gene expression (GSE94752, GSE55200, and GSE48964) and DNA methylation (GSE67024 and GSE111632) datasets were obtained from the GEO database. Differentially expressed genes (DEGs) and differentially methylated genes (DMGs) in subcutaneous adipose tissue of patients with obesity were identified using GEO2R. Methylation-regulated DEGs (MeDEGs) were identified by overlapping DEGs and DMGs. The protein–protein interaction (PPI) network was constructed with the STRING database and analyzed using Cytoscape. Functional modules and hub-bottleneck genes were identified by using MCODE and CytoHubba plugins. Functional enrichment analyses were performed based on Gene Ontology terms and KEGG pathways. To prioritize and identify candidate genes for obesity, MeDEGs were compared with obesity-related genes available at the DisGeNET database.

**Results:**

A total of 54 MeDEGs were identified after overlapping the lists of significant 274 DEGs and 11,556 DMGs. Of these, 25 were hypermethylated-low expression genes and 29 were hypomethylated-high expression genes. The PPI network showed three hub-bottleneck genes (PTGS2, TNFAIP3, and FBXL20) and one functional module. The 54 MeDEGs were mainly involved in the regulation of fibroblast growth factor production, the molecular function of arachidonic acid, and ubiquitin-protein transferase activity. Data collected from DisGeNET showed that 11 of the 54 MeDEGs were involved in obesity.

**Conclusion:**

This study identifies new MeDEGs involved in obesity and assessed their related pathways and functions. These results data may provide a deeper understanding of methylation-mediated regulatory mechanisms of obesity.

## INTRODUCTION

Obesity is a chronic metabolic disorder characterized by an excessive accumulation of body fat resulting from an imbalance between energy intake and expenditure ( 1 , 2 ). Despite nutritional intervention and physical education programs, the prevalence of obesity is increasing in most countries ( 3 ). This disease has been considered the 21st century epidemic ( 1 ) as it affects more than 650 million adults and 124 million children worldwide ( 2 ). Obesity is associated with a number of comorbidities, such as type 2 diabetes mellitus (T2DM), cardiovascular diseases, non-alcoholic fatty liver disease, and some types of cancers, which reduce the quality of life and life expectancy of the affected individuals ( 4 , 5 ).

Obesity is the result of complex and not completely understood pathological processes ( 6 , 7 ). Adipose tissue produces several endocrine factors, cytokines, and chemokines that regulate physiological processes and immune system functions. Moreover, individuals with obesity have increased pro-inflammatory adipokines and chemokines, which contributes to a systemic low-grade chronic inflammation and the development of obesity-related comorbidities ( 8 ). Several agents influence the development of obesity, including environmental factors (especially diet quality and physical activity), gut microbiota composition, endocrine disruptors, and drugs. Moreover, the development of obesity is influenced by a crosstalk between environmental factors, genetic susceptibility, and epigenetic mechanisms ( 9 , 10 ).

Large-scale genome-wide association studies (GWAS) showed more than 500 loci associated with obesity and other related traits ( 11 ). DNA methylation – the transfer of a methyl group to the 5-carbon of cytosine in CpG dinucleotides – is a major epigenetic mechanism that regulates gene expression according to the influence of different environmental factors and hormones ( 6 , 12 , 13 ). Evidence for the role of DNA methylation in obesity has come mostly from animal models ( 1 , 13 , 14 ). Zhang and cols. ( 14 ) identified 178 differentially methylated genes (DMGs) in the liver of mice with high-fat diet (HFD)-induced obesity, showing that HFD changes the epigenetics of hepatocytes and, thus, contributes to the pathophysiology of obesity. In humans, an epigenome-wide DNA methylation association study identified 278 CpG islands associated with variation in body mass index (BMI) in 5,387 individuals ( 15 ).

In the last years, gene expression and epigenome profiling arrays have been used to study thousands of genes for a given disease at the same time. Moreover, integrative bioinformatics analysis of the profiling arrays, using systems biology methodology, emerged as a promising approach to identify and classify differentially expressed genes (DEGs) and DMGs ( 16 , 17 ). Therefore, an integrative analysis using gene expression and DNA methylation datasets of patients with obesity, documented in previous studies, could facilitate the identification of new and potential molecular pathological pathways related to this disease.

Separate DEG and DMG analyses have been used to evaluate genes associated with diseases at different regulation levels. However, by identifying methylation-regulated DEGs (MeDEGs), an integrated network analysis of DNA methylation and gene expression profiling data may provide a deeper understanding of obesity than individual disconnected analyses ( 18 , 19 ). Thus, in this study, we used an integrative analysis approach to identify MeDEGs in the subcutaneous adipose tissue (SAT) from subjects with obesity and assess in which biological functions and pathways they are involved. We implemented a systems biology approach to analyze the protein-protein interaction (PPI) network. Our analysis may provide new ways to understand the mechanisms and pathways underlying obesity.

## MATERIALS AND METHODS

### Microarray data

**Figure 1:**
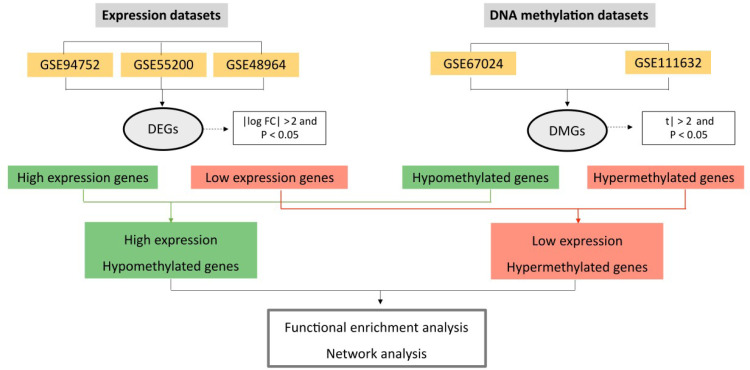
Flowchart of the identification of methylation-regulated differentially expressed genes (MeDEGs) and their function in obesity.

DMG: differentially methylated gene; DEG: differentially expressed gene.

Gene Expression Omnibus (GEO; https://www.ncbi.nlm.nih.gov/geo ) is an international public data repository of microarrays, next-generation sequencing, and other forms of high-throughput functional genomics. The search strategy adopted in GEO was: “obesity” [MeSH terms] AND “Homo sapiens” [Organism] AND (“DNA methylation” OR “Expression profiling” [Filter]). Figure 1 shows that five datasets were retrieved from GEO and included in this study: three of gene expression (GSE94752, GSE55200, and GSE48964) and two of DNA methylation (GSE67024 and GSE111632) analyses in SAT of cases with obesity and lean individuals.

Regarding gene expression datasets, GSE94752 (GPL11532; Affymetrix Human Gene 1.1 ST Array) included isolated adipocytes from abdominal SAT samples from nine lean controls and 21 subjects with obesity ( 20 ). GSE55200 (GPL17692; Affymetrix Human Gene 2.1 ST Array) included SAT samples from seven lean subjects and 16 metabolic healthy patients with obesity ( 21 ). GSE48964 (GPL6244; Affymetrix Human Gene 1.0 ST Array) analyzed gene expression in adipose-derived stem cells (hASCs) from three patients with severe obesity and three individuals without obesity ( 22 ).

Regarding DNA methylation datasets, GSE67024 (GPL13534; Illumina HumanMethylation450 BeadChip) analyzed SAT from 15 women with obesity and 14 lean women ( 23 ) and GSE111632 (GPL13534; Illumina HumanMethylation450 BeadChip) included hASCs isolated from six women with obesity and six lean women ( 24 ).

### Data processing

GEO2R ( http://www.ncbi.nlm.nih.gov/geo/geo2r/ ) is an interactive web tool that allows users to compare two or more groups of samples in a GEO Series. This tool was used to analyze the selected datasets and identify DMGs and DEGs in SAT of individuals with obesity compared with lean controls. When a gene symbol corresponded to multiple probe IDs, the average value of these probes was estimated as the representative expression level of this gene. Gene identifiers were mapped according to the HUGO Gene Nomenclature Committee (HGNC) ( 25 ) and only valid identifiers were maintained.

DEGs were defined based on an absolute log2-Fold Change (log FC) > 2.0 and P < 0.05. To identify DMGs, |t|>2 and P < 0.05 were used as cutoff points. Using the Excel LOOKUP function (VLOOKUP), datasets were overlapped according to the expression/methylation profile. Hypomethylated-high expression genes were identified after overlapping upregulated and hypomethylated genes and hypermethylated-low expression genes were identified after overlapping downregulated and hypermethylated genes ( Figure 1 ). Hypomethylated-high expression genes and hypermethylated-low expression genes were identified as MeDEGs. Results were presented in Venn diagrams, which were constructed in the InteractiveVenn website ( 26 ).

### Protein-protein interaction network

The PPI network of obesity-related MeDEGs was constructed using the free web-available Search Tool for the Retrieval of Interacting Genes (STRING version 11.0; http://string-db.org/ ) database ( 27 ). An interaction score > 0.4 and P < 0.05 were considered as statistically significant. Results from STRING were imported into Cytoscape 3.8.1 ( 28 ) for the network analysis and visualization.

### Systems biology approach to analyze the PPI network based on MeDEGs

Complex biological systems may be represented and analyzed as computable networks. Nodes and edges are the basic components of a network. Nodes are connected by edges (which are also called links or lines) and edges show the relationships between nodes ( 29 ). In this study, the nodes were the genes and the edges were lines showing the interaction force between them, thus forming a PPI network.

The relevance of each node for the network was assessed by two centrality measures: degree and betweenness. Degree quantifies the number of connections from a given node ( 30 ). Highly connected nodes are called hubs and tend to be important control points in the network. Betweenness is the number of minimum non-redundant paths between two nodes that cross a given node ( 31 ). Nodes with high betweenness are called bottlenecks and tend to act as major intersections between modules in networks ( 27 , 32 ). In this study, hubs and bottlenecks were defined as nodes in the top 10% of degree and betweenness distributions ( 33 ), respectively, with a minimum of two interactions. Hub and bottleneck genes were identified using the CytoHubba plugin version 0.1 ( 34 ) for Cytoscape.

Cluster analysis to identify modules within the PPI network was performed using the Molecular Complex Detection (MCODE) version 2.0.0 plugin ( 35 ) and considering a number of nodes > 3.0 as a cutoff point. This algorithm identifies in the PPI network densely connected regions that are likely to represent functional interaction complexes, since proteins in the same cluster tend to enrich common biological functions ( 36 ).

### Functional enrichment analysis

The Gene Ontology (GO) analysis is extensively used to identify the characteristic biological attributes of genes, gene products, and sequences, including biological processes (BP), cell components (CC), and molecular function (MF) ( 37 ). The Kyoto Encyclopedia of Genes and Genomes (KEGG) is a collection of databases on genomes, biological pathways, diseases, and chemical substances ( 38 ). In this study, GO terms and KEGG pathway enrichment analyses were performed using STRING version 11.0. A hypergeometric test was used to estimate the statistical significance of enriched pathways and P-values were corrected for multiple tests using the Benjamini-Hochberg procedure, which provides a false discovery rate (FDR)-adjusted-P-value (q-value). GO terms and KEGG pathways associated with q < 0.05 were considered significantly enriched.

### MeDEG candidate selection

To prioritize and identify candidate genes for obesity, the DisGeNET database ( http://www.disgenet.org ) was used. The obesity-related MeDEGs identified in this study were compared with experimentally validated and computationally predicted obesity-related genes (C0028754), according to DisGeNET. This database is a comprehensive platform that integrates information on human disease-associated genes and includes data from expert curated repositories, text mining data extracted from scientific literature, experimentally validated data, and referred data ( 39 , 40 ).

## RESULTS

### DMGs and DEGs in obesity

**Figure 2:**
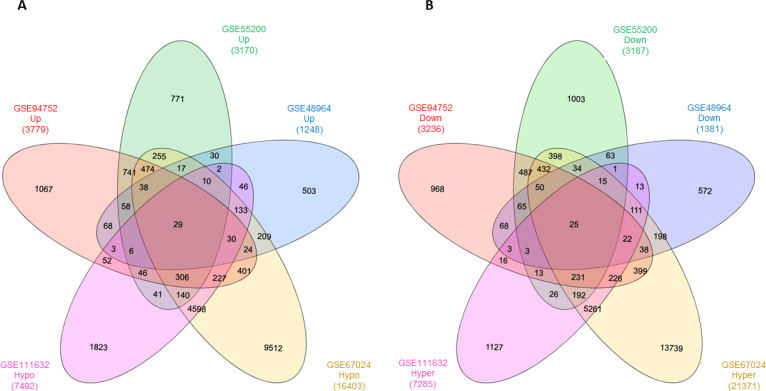
Identification of methylation-regulated differentially expressed genes (MeDEGs) by overlapping gene expression datasets (GSE94752, GSE55200, and GSE48964) and DNA methylation datasets (GSE67024 and GSE111632) in the subcutaneous adipose tissue of subjects with obesity. ( **A** ) Hypomethylated (hypo)-high (up) expression genes; ( **B** ) Hypermethylated (hyper)-low (down) expression genes.

Figure 1 presents the flowchart of the analysis strategy used in this study. Regarding DEGs, we identified 131 upregulated and 143 downregulated genes after overlapping the three gene expression datasets. Regarding DMGs, we identified 6,083 hypermethylated and 5,473 hypomethylated genes after overlapping the two datasets. After overlapping the five datasets, we identified 29 hypomethylated-high expression ( Figure 2A ) and 25 hypermethylated-low expression genes ( Figure 2B ), totalizing 54 MeDEGs.

### PPI network construction, module analysis, and identification of hub and bottleneck genes

**Figure 3:**
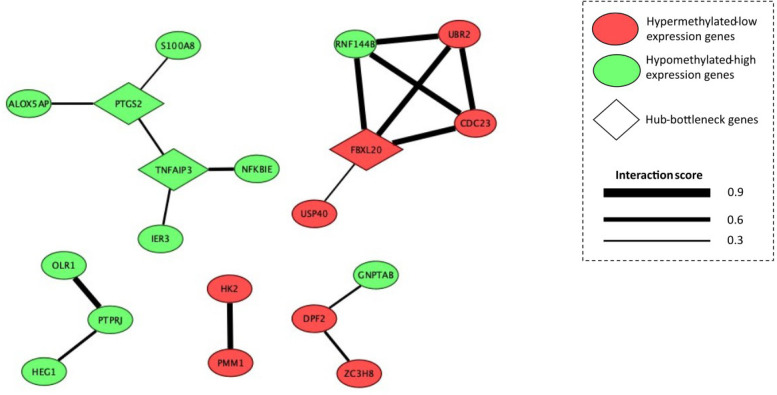
PPI network formed by 19 MeDEGs: protein-protein interaction (PPI). Green nodes represent hypomethylated-high expression genes in obesity and red nodes represent hypermethylated-low expression genes. Diamond nodes represent the hub-bottleneck genes identified in the network analysis. Nodes represent the proteins encoded by each gene and the edges represent the protein-protein interactions. Interaction score refers to “combined score” in the STRING database. The thicknesses of the edges was associated with the combined scored. This score ranges from 0.0 (weak interaction) to 1.0 (strong interaction).

The PPI network was constructed in the STRING database and includes 19 nodes (MeDEGs) and 17 edges (connecting lines) ( Figure 3 ). The nodes represent the proteins encoded by each gene and the edges represent protein-protein interactions. Of the 54 MeDEGs, 35 had no interconnection within the network, thus, we excluded them from the further analysis. Among the 19 MeDEGs present in the network, 11 were hypomethylated-high expression genes and eight were hypermethylated-low expression genes. Moreover, the highest interaction score (0.9) was between *RNF144B* , *UBR2* , *CDC23* , and *FBXL20* , and between *HK2* and *PMM1* ( Figure 3 ).

**Figure 4:**
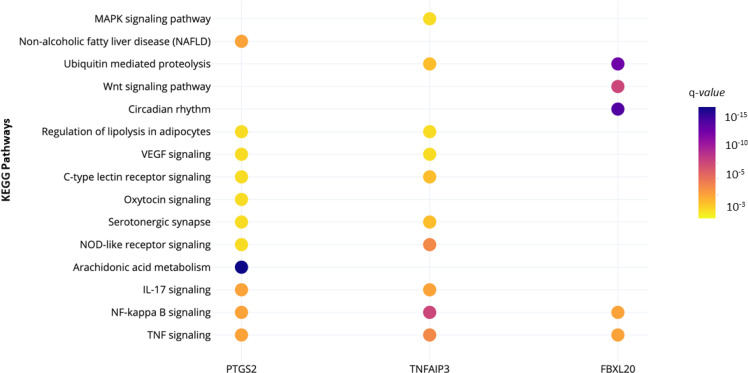
Significant KEGG pathways in which participate each hub-bottleneck gene. The color of the circle represents statistical significance, according to the q-value.

We used two algorithms to measure centrality in the PPI network: degree (hub genes) and betweenness (bottleneck genes). We considered the three most connective nodes as hub-bottleneck genes: prostaglandin-endoperoxide synthase 2 ( *PTGS2* ), tumor necrosis factor α-induced protein 3 ( *TNFAIP3* ), and F-box and leucine rich repeat protein 20 ( *FBXL20* ), which might play a critical role in obesity. These three genes participate in several KEGG pathways, including TNF, NF-κB, IL-17, ubiquitin mediated proteolysis, Wnt signaling pathway, and the regulation of lipolysis in adipocytes ( Figure 4 ).

**Figure 5:**
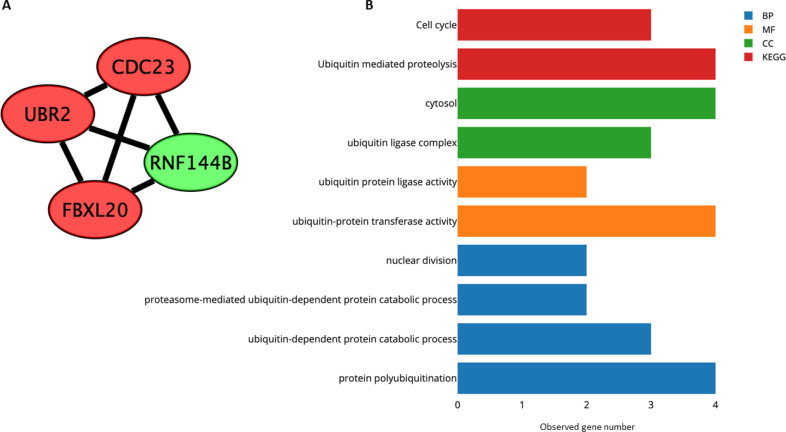
Module identification within the PPI network. ( **A** ) Significant module identified by MCODE. Green nodes represent hypomethylated-high expression genes in obesity and red nodes represent hypermethylated-low expression genes. ( **B** ) Significant GO terms and KEGG pathways in which genes participate. The y-axis represents GO terms or KEGG pathways and the x-axis refers to the number of genes enriched on GO terms or KEGG pathways.

We performed the further analysis of the PPI network to identify the most significantly connected and tightly clustered subnetworks. We selected from the PPI network a significant module using MCODE ( Figure 5A ). In this module, we identified four genes ( *CDC23* , *FBXL20* , *RNF144B* , and *UBR2* ). This module was mainly enriched for cell cycle and ubiquitin mediated proteolysis signaling pathways. Regarding GO terms, these genes were enriched in biological processes and molecular functions related to ubiquitination and nuclear division ( Figure 5B ).

### Functional enrichment analysis of MeDEGs

Table S1 presents the full results of the GO terms and KEGG pathways enrichment analysis for the 54 MeDEGs. Results showed that the 29 hypomethylated-high expression genes were involved in biological processes of positive regulation of fibroblast growth factor (FGF) production. Regarding molecular function, MeDEGs were also related to arachidonic acid binding. The 25 hypermethylated-low expression genes were not significantly enriched in GO terms.

**Supplementary Table 1:** Gene ontology and KEGG pathways of the 54 MeDEGs broken down by their methylation/expression profile

	54 MeDEGs together	
**GENE ONTOLOGY**		
	**Biological Process**	
GO term	Description	FDR
GO:0090270	regulation of fibroblast growth factor production	0.0042
	**Molecular Function**	
GO term	Description	FDR
GO:0050544	arachidonic acid binding	0.0379
GO:0004842	ubiquitin-protein transferase activity	0.0379
No significant KEGG Pathway was found for these set of genes		
**29 Hypomethylated-highly expressed MeDEGs**		
**GENE ONTOLOGY**		
	**Biological Process**	
GO term	Description	FDR
GO:0090271	positive regulation of fibroblast growth factor production	0.0385
	**Molecular Function**	
GO term	Description	FDR
GO:0050544	arachidonic acid binding	0.0073
**KEGG Pathway**		
Pathway	Pathway Name	FDR
hsa04657	IL-17 signaling	0.0034
**25 Hypermethylated-low expressed MeDEGs**		
**KEGG Pathway**		
Pathway	Pathway Name	FDR
hsa00051	Fructose and mannose metabolism	0.0408
hsa00520	Amino sugar and nucleotide sugar metabolism	0.0415
hsa00310	Lysine degradation	0.0415
hsa00010	Glycolysis / Gluconeogenesis	0.0415

FDR: False Discovery Rate. Method: Benjamini-Hochenberg.

Regarding KEGG pathways, the 29 hypomethylated-high expression genes participate especially in the IL-17 signaling. The 25 hypermethylated-low expression genes participate in fructose and mannose metabolism, amino sugar and nucleotide sugar metabolism, lysine degradation, and glycolysis/gluconeogenesis pathways.

All 54 MeDEGs were mainly involved in biological processes of regulation of FGF production ( Table S1 ). Moreover, these MeDEGs were related to the molecular function of arachidonic acid and ubiquitin-protein transferase activity ( Table S1 ).

### Comparison between the 54 MeDEGs and obesity-related genes identified in the DisGeNET database

To identify key obesity-related genes, we accessed the DisGeNET database. Of the 54 MeDEGs identified in our study, 11 were also identified in the search in DisGeNET ( *ALDH2* , *ALOX5AP* , *BAMBI* , *DOCK5* , *FUZ* , *HK2* , *LRRFIP1* , *OLR1* , *PTGS2* , *PTPRJ* , and *S100A8* ) ( Table 1 ). Moreover, of the three hub-bottleneck genes ( *PTGS2* , *TNFAIP3* , and *FBXL2* ) *,* only *PTGS2* was obesity-related, according to DisGeNET. Thus, *FBXL20* and *TNFAIP3* could be new candidate genes to be studied in the context of obesity.

**Table 1:** Comparison between the list of 54 MeDEGs and those genes related to obesity in the DisGeNet database

Gene symbol	Gene name	Group	DisGeNet	Other genes from the same gene family that were associated with obesity according to DisGeNet
*ALDH2*	Aldehyde dehydrogenase 2 family member	Hiper/Low	Yes	*ALDH1L1, ALDH1A1, ALDH6A1*
*ALOX5AP*	Arachidonate 5-lipoxygenase activating protein	Hypo/Highly	Yes	*ALOX12, ALOX15, ALOX5*
*ANKRD44*	Ankyrin Repeat Domain 44	Hypo/Highly	No	*ANKK1, ANKRD26*
*AOAH*	Acyloxyacyl Hydrolase	Hypo/Highly	No	
*BAMBI*	BMP and activin membrane bound inhibitor	Hypo/Highly	Yes	
*C12orf75*	Chromosome 12 Open Reading Frame 75	Hypo/Highly	No	
*CACHD1*	Cache Domain Containing 1	Hyper/Low	No	
*CD9*	CD9 Molecule	Hypo/Highly	No	*CD14, CD163, CD180, CD1D, CD200, CD24, CD248, CD274, CD33, CD36, CD38, CD40, CD44, CD47, CD48, CD59, CD5L, CD68, CD69, CD74, CD79A, CD81, CD8A*
*CDC23*	Cell Division Cycle 23	Hyper/Low	No	*CDC42*
*COL14A1*	Collagen Type XIV Alpha 1 Chain	Hypo/Highly	No	*COL12A1, COL1A1, COL25A1, COL4A1, COL6A1, COL6A3, COL9A3*
*DOCK5*	Dedicator of cytokinesis 5	Hypo/Highly	Yes	*DOCK2*
*DPF2*	Double PHD fingers 2	Hyper/Low	No	
*ECHDC2*	Enoyl-CoA hydratase domain containing 2	Hyper/Low	No	*ECHS1*
*EZH1*	Enhancer of zeste 1 polycomb repressive complex 2 subunit	Hyper/Low	No	*EZH2*
*FAM105A/OTULINL*	OTU deubiquitinase with linear linkage specificity like	Hypo/Highly	No	*FAM13A, FAM161A, FAM3A, FAM3B, FAM71F1*
*FBXL20*	F-box and leucine rich repeat protein 20	Hyper/Low	No	*FBXO3*
*FGD6*	FYVE, RhoGEF And PH Domain Containing 6	Hypo/Highly	No	
*FUZ*	Fuzzy planar cell polarity protein	Hyper/Low	Yes	
*GNPTAB*	N-acetylglucosamine-1-phosphate transferase Subunits alpha and beta	Hypo/Highly	No	
*HEG1*	Heart development protein with EGF like domains 1	Hypo/Highly	No	
*HK2*	Hexokinase 2	Hyper/Low	Yes	*HK1*
*IER3*	Immediate early response 3	Hypo/Highly	No	
*IKZF1*	IKAROS family zinc finger 1	Hypo/Highly	No	
*LRRC1*	Leucine rich repeat containing 1	Hypo/Highly	No	*LRPPRC, LRRC53, LRRC8A*
*LRRFIP1*	LRR binding FLII interacting protein 1	Hypo/Highly	Yes	*LRRN1, LRRN4*
*MCOLN3*	Mucolipin TRP cation channel 3	Hyper/Low	No	
*MICALL2*	MICAL like 2	Hypo/Highly	No	
*NFKBIE*	NFKB inhibitor epsilon	Hypo/Highly	No	*NFKB1*
*NTN4*	netrin 4	Hypo/Highly	No	
*NUMA1*	Nuclear mitotic apparatus protein 1	Hyper/Low	No	
*OLR1*	Oxidized low density lipoprotein receptor 1	Hypo/Highly	Yes	
*PIK3AP1*	Phosphoinositide-3-kinase adaptor protein 1	Hypo/Highly	No	
*PMM1*	Phosphomannomutase 1	Hyper/Low	No	
*PTGS2*	Prostaglandin-endoperoxide synthase 2	Hypo/Highly	Yes	*PTGS1*
*PTPRJ*	Protein tyrosine phosphatase receptor type J	Hypo/Highly	Yes	*PTPRB, PTPRC, PTPRE, PTPRF, PTPRS, PTPRU*
*RHOXF1*	Rhox homeobox family member 1	Hypo/Highly	No	
*RNF144A*	Ring finger protein 144A	Hyper/Low	No	*RNF19A, RNF216, RNF41*
*RNF144B*	Ring finger protein 144B	Hypo/Highly	No	*RNF19A, RNF216, RNF41*
*S100A8*	S100 calcium binding protein A8	Hypo/Highly	Yes	*S100A12, S100A16, S100A4, S100A6, S100A7, S100A8, S100A9, S100B*
*SLC25A38*	Solute carrier family 25 member 38	Hyper/Low	No	*SLC10A1, SLC12A3, SLC12A9, SLC15A1, SLC16A1, SLC16A7, SLC17A5, SLC19A1, SLC22A1, SLC22A12, SLC22A2, SLC22A3, SLC22A6, SLC22A8, SLC24A3, SLC25A19, SLC25A3, SLC27A1, SLC27A2, SLC2A5, SLC27A4, SLC27A5, SLC27A6, SLC2A1, SLC2A12, SLC2A2, SLC2A3, SLC2A4, SLC2A9, SLC30A10, SLC30A8, SLC33A1, SLC35A1, SLC35B2, SLC35B4, SLC35D3, SLC35G1, SLC37A4, SLC38A1, SLC38A2, SLC38A5, SLC39A13, SLC45A2, SLC4A4, SLC5A1, SLC5A11, SLC5A2, SLC5A5, SLC6A12, SLC6A14, SLC6A2, SLC6A3, SLC6A4, SLC6A8, SLC7A14, SLC8A1, SLC9A3, SLC9A6, SLC9A7*
*SLC36A4*	Solute Carrier Family 36 Member 4	Hypo/Highly	No	
*SPON1*	Spondin 1	Hyper/Low	No	
*STX17*	Syntaxin 17	Hyper/Low	No	*STX16, STX8*
*TMTC2*	Transmembrane O-Mannosyltransferase Targeting Cadherins 2	Hypo/Highly	No	*TMTC1*
*TNFAIP3*	TNF Alpha Induced Protein 3	Hypo/Highly	No	–
*TTC21B*	Tetratricopeptide Repeat Domain 21B	Hyper/Low	No	*TTC28, TTC28-AS1, TTC8*
*TXNIP*	Thioredoxin interacting protein	Hyper/Low	No	*TXNRD2, TXNRD3*
*UBR2*	Ubiquitin protein ligase E3 component n-recognin 2	Hyper/Low	No	–
*UBXN7*	UBX domain protein 7	Hyper/Low	No	–
*UNC119B*	Unc-119 lipid binding chaperone B	Hyper/Low	No	–
*USP40*	Ubiquitin specific peptidase 40	Hyper/Low	No	*USP10, USP17L2, USP17L24, USP17L25, USP17L26, USP17L27, USP17L28, USP17L29, USP17L30, USP17L9P, USP19, USP27X, USP8, USP9X*
*WDR75*	WD repeat domain 75	Hyper/Low	No	*WDR11, WDR26*
*WNT11*	Wnt family member 11	Hyper/Low	No	*WNT1, WNT10B, WNT3A, WNT4, WNT5A, WNT5B, WNT7A*
*ZC3H8*	Zinc finger CCCH-type containing 8	Hyper/Low	No	

Hypo: hypomethylated gene; Hyper: hypermethylated gene; Low: low expressed gene; Highly: hightly expressed gene.

## DISCUSSION

Previous studies have mainly focused on the association between the expression of individual genes and DNA methylation profiles in the context of obesity. However, bioinformatics analyses allow the combination of data from a large number of DEGs and DMGs and thus the identification of key genes that are simultaneously differentially expressed and methylation-regulated ( 18 ). Regarding obesity, studies showing the interaction between genes and epigenetic factors are specially interesting, as several environmental factors influence the development of this disease.

In this study, we identified 54 MeDEGs, of which 29 are hypomethylated-high expression genes and 25 are hypermethylated-low expression genes. Functional enrichment analysis showed that these MeDEGs participate in many biological processes and molecular functions, including the positive regulation of FGF production, arachidonic acid binding, and ubiquitin-protein transferase activity.

FGFs are secreted signaling proteins with various functions in cell proliferation, development, and wound healing ( 41 , 42 ). In the last years, several FGFs have been involved in the regulation of glucose and lipid metabolism ( 43 ) and FGF1, FGF19, and FGF21 are the main factors associated with energy metabolism ( 43 , 44 ). Evidence from studies with mice showed that Fgf1 is involved in the expansion of adipose tissue during HFD feeding, which suggests that this protein promotes preadipocyte proliferation and differentiation ( 45 ). In accordance with these findings, individuals with obesity have higher FGF1 secretion in SAT compared with lean individuals ( 46 ). The beneficial effects of FGF19 and FGF21 on metabolism are more established than those of other members of this family. These two proteins increase energy expenditure and decrease body weight, glucose intolerance, and blood glucose ( 43 ). Thus, FGFs and their derivatives might have great potential as new therapies to treat metabolic conditions ( 47 ).

Regarding the enrichment of MeDEGs in arachidonic acid binding, few studies reported arachidonic acid acts as a precursor of pro-inflammatory metabolites, including prostaglandin E2, leukotrienes, hydroxy-eicosatetraenoic acids, and diacylglycerol, which are molecules also involved in insulin resistance ( 48 ). Thus, arachidonic acid may also contribute to obesity. MeDEGs were also enriched in ubiquitin-protein transferase activity, which seems to have significant effects on obesity, mainly due to its involvement in inflammation, cholesterol, and glucose metabolism ( 49 ). Our results provide new information to understand obesity.

The PPI network analysis identified three hub-bottleneck genes: *PTGS2* , *TNFAIP3* , and *FBXL20* . Hubs and bottlenecks are crucial components in signaling networks, as they are relevant genes for biologically significant processes in the disease development ( 32 , 48 ). *PTGS2* and *TNFAIP3* were identified as hypomethylated-high-expression genes and *FBXL20* as a hypermethylated-low expression gene in SAT of individuals with obesity. Moreover, functional enrichment analysis showed that these three hub-bottleneck genes play an essential role in several signaling pathways, including TNF, NF-κB, IL-17, ubiquitin mediated proteolysis, Wnt, and the regulation of lipolysis, suggesting that these genes participate in obesity-related pathways and could be essential to understand the pathophysiology of obesity.

*TNFAIP3* encodes TNFAIP3/A20, which is a ubiquitin-modifying enzyme ( 50 , 51 ). *TNFAIP* 3 negatively regulates NF-κB activity, which mediates the effects of pro-inflammatory cytokines, including TNF and IL-1β ( 52 , 53 ). In mice, the overexpression of *TNFAIP3* inhibits NLRP3 inflammasome complex, preventing lupus inflammation and renal injury ( 53 ). NLRP3 is negatively associated with obesity due to increased inflammation in adipose tissue ( 54 ). Accordingly, Rendo-Urteaga and cols. ( 55 ) observed a downregulation of *TNFAIP3* in high-responder children with obesity to a dietary intervention program, possibly leading to a better inflammatory state by decreased TNF secretion.

PTGSs are rate-limiting enzymes in the conversion of arachidonic acid, a product of damaged cell membranes, into prostaglandins ( 56 ). The expression of the *PTGS2* isoform ( *COX-2* ) is induced by IL-1β, IL-6, and TNF; thus, it is upregulated during inflammation ( 56 ). In obesity, adipocyte *COX-2* activation seems to upregulate the macrophage migration inhibitory factor (MIF) production via NF-κB activation. In turn, during early stages of inflammation, MIF secretion by adipocytes recruits pro-inflammatory macrophages (M1), leading to the secretion of several pro-inflammatory cytokines, which characterizes a feedback cycle that results in chronic inflammation associated with insulin resistance in obesity ( 57 ).

FBXL20 is an E3 ubiquitin ligase that plays a key role in ubiquitin-mediated proteolysis. Evidence show that FBXL2, a protein of the same family, preserves cardiac homeostasis in the face of HFD-induced obesity ( 58 ). However, FBXL20 has not yet been associated with obesity or its related comorbidities. Thus, further studies are needed to assess its role in the development of obesity.

Further analysis of the PPI network using MCODE showed a module with the highest interaction between nodes, including four genes ( *CDC23* , *FBXL20* , *UBR2* , and *RNF144B* ). These genes were enriched for cell cycle and ubiquitin-mediated proteolysis, which is the major pathway for regulated degradation of intracellular proteins to prevent the accumulation of damaged proteins ( 59 ). The upregulation of *FTO* has been robustly associated with increased BMI and predisposition to obesity and T2DM ( 60 ). Zhu and cols. ( 61 ) showed that *FTO* undergoes active ubiquitination on the evolutionarily conserved Lys-216 residue, which leads *FTO* to proteasomal degradation. This might have a protective effect on energy metabolism, food intake regulation, fat metabolism, and body weight. Taking that into account, the lack of ubiquitin-mediated proteolysis could potentially lead to obesity.

Comparing our results with data available at DisGeNET, 11 MeDEGs identified in our study were also previously associated with obesity in the database. Interestingly, our study also suggested other genes that have a role in obesity that were not included in the database. Therefore, this set of genes might play an important role in the development/progression of obesity.

Although we identified potential candidate genes for obesity using bioinformatics techniques, our study has a few limitations. First, the DNA methylation and gene expression datasets had relatively small sample sizes, which might limit the application of our findings. Moreover, the samples of all DNA methylation datasets and one gene expression dataset (GSE94752) had only women, which might limit the application of our findings for men. Second, we analyzed both DNA methylation and gene expression only in SAT; thus, the results could be different if we had analyzed in other tissues. Lastly, we did not validate our bioinformatics data in SAT samples from patients with obesity and lean individuals due to unavailability of samples. Thus, further studies are needed to confirm our data. Despite these limitations, our findings are important to understand the complex regulation of gene expression in obesity and present new potential targets genes and pathways.

In conclusion, we mapped 54 MeDEGs possibly related to obesity by combined gene expression and DNA methylation profile data analyses. By a series of bioinformatics analyses, we identified hub-bottleneck genes and key pathways involved in obesity. We also presented a list of candidate genes for obesity using DisGeNET. These results may deepen the understanding of epigenetic regulation mechanisms involved in the development/progression of obesity. Finally, molecular biological experiments are needed to confirm the function of the identified genes in obesity and their interactions.
